# A score of non-contrast transthoracic echocardiography to screen patent foramen ovale in patients with embolic stroke of undetermined source

**DOI:** 10.1186/s12883-022-02565-w

**Published:** 2022-02-04

**Authors:** Hui Zhang, Haiyan Tang, Fei Wu, Chun Yu, Qiang Dong, Wenjie Cao

**Affiliations:** 1 Department of Neurology, Huashan Hospital, Fudan University, No.12 Wulumuqi Zhong Rd, Shanghai, 200040 China; 2 Department of Neurology and Institute of Neurology, Shuguang Hospital Affiliated to Shanghai University of Traditional Chinese Medicine, Shanghai, China; 3 Department of Critical Care, West Campus of Huashan Hospital, Fudan University, Shanghai, China; 4 State Key Laboratory of Medical Neurobiology, Fudan University, Shanghai, China; 5 National Clinical Research Center for Aging and Medicine, Huashan Hospital, Fudan University, Shanghai, China

**Keywords:** Transthoracic echocardiography, Patent foramen ovale, Embolic stroke of undetermined source, Bubble test, Transcranial Doppler

## Abstract

**Background:**

The aim of this study was to develop a screening score system of non-contrast transthoracic echocardiography (TTE) for patent foramen ovale (PFO) in patients with embolic stroke of undetermined source (ESUS).

**Methods:**

We performed a retrospective analysis of 218 consecutive patients with a recent ESUS from 2015 to 2018, who received TTE and transcranial Doppler (TCD) as routine examinations. PFO was diagnosed by the bubble test of TCD. Significant differences of the non-contrast TTE findings and patient characteristics between PFO group and non-PFO group were selected into a score.

**Results:**

PFO was diagnosed in 35.8% (78/218) of the patients. Compared with non-PFO group, a larger median aortic root diameter (ARd) (34 mm vs. 32 mm, *p* = 0.005), a lower median peak E wave velocity (Em) (61.5 cm/s vs. 68 cm/s, *p* = 0.005) and a lower incidence rate of mitral regurgitation (34.6% vs. 50.7%, *p* = 0.022) were seen in PFO group. ARd>33 mm and Em < 72 cm/s were the best thresholds to predict PFO in ROC analysis. A four-point score system (MEAD) including TTE criteria (including ARd>33 mm, Em < 72 cm/s and without mitral regurgitation) and no history of diabetes predicted PFO with an area under curve of 0.67 (95%CI 0.57–0.72, *p* < 0.001). MEAD score ≥ 3 was the best threshold to predict PFO with an accuracy of 0.64 (95% CI 0.57–0.7), a sensitivity of 0.65 (95% CI 0.53–0.75) and a specificity of 0.63 (95% CI 0.55–0.71).

**Conclusion:**

The MEAD score measured with non-contrast TTE can be used to select patients for bubble test of TCD to increase the diagnostic yield of PFO after ESUS.

**Supplementary Information:**

The online version contains supplementary material available at 10.1186/s12883-022-02565-w.

## Background

Embolic stroke of undetermined source (ESUS), which accounts for approximately 25% of cases and patent foramen ovale (PFO) is considered to be one of the major causes of ESUS [1]. Ultrasonographic assessment of PFO, using bubble test with transthoracic echocardiograph (TTE), transesophageal echocardiography (TEE) and transcranial Doppler (TCD) remains the diagnostic approach of choice [2]. Although the prevalence of cerebrovascular complications after bubble test were reported very low, stroke risk from paradoxical microbubble embolization can be clinically significant and cannot be guaranteed [3]. Additionally, TTE and bubble test with TCD or TTE are still not routine examinations in most of the stroke centers. Thus, a safe and useful PFO-screening tool before contrast ultrasonographic assessment is important for clinical practice. In this study, we aimed to develop an easily measured screening tool by using non-contrast TTE for PFO in patients with ESUS.

## Methods

### Patients

We retrospectively reviewed patients diagnosed with acute ESUS from Jan 2015 to Jun 2018. ESUS was defined according to TOAST (Trial of Org 10,172 in Acute Stroke Treatment) classification. Those with data of both TTE and bubble test on TCD were included. Demographic characteristics, vascular risk factors (diabetes, hypertension, hyperlipidemia, smoking) and previous stroke history were documented. Ten-point Risk of Paradoxical Embolism (RoPE) score was calculated [4]. Brain magnetic resonance imaging or CT scan were reviewed to determine the locations of infarcts.

### Bubble test on TCD

PFO was diagnosed by the bubble test on TCD [5]. TCD was done according to the Consensus Conference of Venice15 by one of two experienced operators. A head frame was placed on the head to maintain the two 2 MHz probes in place for monitoring bilateral middle cerebral artery (MCA) at a depth of 50-60 mm. 9 mL isotonic saline solution, 1 mL of air, and 1 drop of the patient’s blood were mixed through two 10 mL syringes connected by a three-way stopcock. The mixture was injected at least twice to assess the presence of microbubbles (MBs) in either MCA during the TCD monitoring. A first bolus was injected during normal respiration with the patient at rest. The procedure was repeated after a performance of strenuous Valsalva Maneuver by the patient. Right-to-left shunt (RLS) was quantified by counting the number of MBs within the first 3 cardiac cycles. More than 1 MBs detected over a 25-s post injection interval was considered to be positive contrast-enhanced TCD [6].

### TTE

Parameters of TTE were obtained by retrospectively reviewing the reports. TTE were performed in the left lateral decubitus position using standard imaging planes according to the American Society of Echocardiography recommendations during hospitalization. The aorta root diameters (ARd) and left atria anteroposterior diameters (LAAPd), end-diastolic left ventricle diameters (EDLVd), end-systolic left ventricle diameters (ESLVd), left ventricle ejection fraction (LVEF), peak E-wave velocity (Em) and peak A-wave velocity (Am) were collected. Presences of mitral, tritral and aortic valve regurgitation were recorded.

### Statistical Analysis

Statistical analyses were performed using SPSS, version 22 (SPSS Inc., Chicago, IL) and R software (https://www.r-project.org). *P* value less than 0.05 was considered to indicate statistical significance. Continuous variables were compared by Mann–Whitney U test and categorical variables were compared by Chi-square or Fisher’s exact test between PFO and non-PFO groups. Multivariate regression including remarkable TTE findings and 10 individual items of RoPE score (including patient characteristics and age grades of per 10-year increase) was used to access the association of variables with PFO. Receiver operating characteristic (ROC) analysis was performed to determine the optimal threshold of remarkable continuous variables in predicting PFO. Base on the results, a 4-point score, including 4 independent criteria, was tested by ROC for predicting PFO. A 10-fold cross validation of the 4-score system was performed by using R software.

## Results

**Fig. 1:**
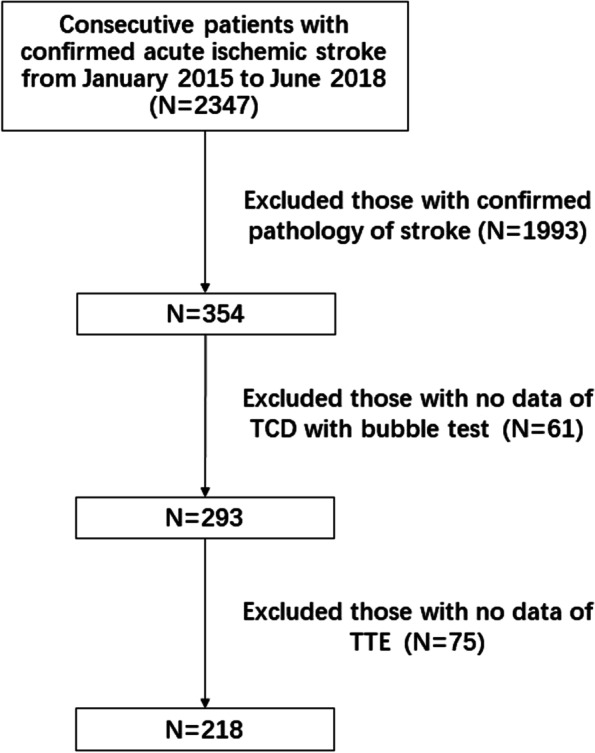
Flowchart of patient selection

A total of 218 ESUS patients were finally included, (Fig. 1) median age was 53 years (interquartile range [IQR], 39.75–60), 74.8% were male (163/218). PFO was detected in 35.8% (78/218) of the patients. No difference was found in age, sex, risk factors for stroke, infarct patten and RoPE score between PFO group and non-PFO group. In comparation of TTE parameters, a larger median ARd (measured at the level of the sinuses of Valsalva) (34 mm vs. 32 mm, p = 0.005), lower median Em (61.5 cm/s vs. 68 cm/s, *p* = 0.005) and lower incidence of mitral regurgitation (34.6% vs. 50.7%, *p* = 0022) were found in the PFO group. (Table 1).

**Table 1:** Univariate analysis between PFO group with non-PFO group

Variable	Total *n* = 218	PFO *n* = 78	Non-PFO *n* = 140	*P* value
Age, yr, median (IQR)	53 (39.75, 60)	52.5 (43, 59.25)	53 (38.25, 61)	0.846
Sex, male, % (n)	74.8 (163)	82.1 (64)	70.7 (99)	0.065
*Clinical characteristics*				
Diabetes, % (n)	18.8 (41)	12.8 (10)	22.1 (31)	0.091
Hypertension, % (n)	40.4 (88)	42.3 (33)	39.3 (55)	0.663
Hyperlipidemia, % (n)	17.4 (38)	17.9 (14)	17.1 (24)	0.88
Smoking, % (n)	39.4 (86)	39.7 (31)	39.3 (55)	0.947
Previous TIA/stroke, % (n)	17.4 (38)	16.7 (13)	17.9 (25)	0.824
Cortical infarcts, % (n)	55.5 (121)	60.3 (47)	52.9 (74)	0.292
*RoPE score*				
RoPE score, median (IQR)	5 (4, 7.25)	6 (4, 7)	5 (4, 8)	0.848
RoPE score >6, % (n)	36.2 (79)	30.8 (24)	39.3 (55)	0.21
*TTE parameters*				
ARd, mm, median (IQR)	33 (31, 36)	34 (31, 36.25)	32.5 (30, 35)	0.005
LAAPd, mm, median (IQR)	36 (33, 38)	36 (33, 38)	36 (33, 38)	0.961
EDLVd, mm, median (IQR)	49 (46, 52)	48 (45, 53)	49 (46, 52)	0.759
ESLVd, mm, median (IQR)	30 (28, 32)	30 (28, 32)	30 (27, 32)	0.961
LEVF, %, median (IQR)	69 (65, 72)	69 (66, 72)	69 (65.25, 72)	0.814
Em, cm/s, median (IQR)	66 (53, 77.5)	61.5 (51, 70.25)	68 (56, 82)	0.005
Am, cm/s, median (IQR)	68 (55, 80)	67.5 (55.5, 79)	68.5 (55, 80)	0.263
Mitral regurgitation, % (n)	45 (98)	34.6 (27)	50.7 (71)	0.022
Tritral regurgitation, % (n)	19.7 (43)	19.2 (15)	20 (28)	0.891
Aortic valve regurgitation, % (n)	20.6 (45)	23.1 (18)	19.3 (27)	0.507

*PFO* patent foramen ovale, *IQR* interquartile range, *ARd* aortic root diameter, *RoPE* Risk of Paradoxical Embolism, *TTE* transthoracic echocardiography, *LAAPd* left atria anteroposterior diameters, *EDLVd* end-diastolic left ventricle diameters, *ESLVd* end-systolic left ventricle diameters, *LVEF* left ventricle ejection fraction, *Em* peak E wave velocity, *Am* peak A wave velocity

In ROC analysis, ARd predicted PFO with an area under curve (AUC) of 0.61 (95%CI 0.53–0.69, *p* = 0.005) (Fig. 1, in Supplement) and Em predicted PFO also with an AUC of 0.61 (95%CI 0.53–0.69, *p* = 0.005) (Fig. 2, in Supplement). Threshold of ARd and Em in predicting PFO was 33 mm and 72 cm/s (analyzed with Youden index). In multivariate regression analysis, ARd >33 mm, Em < 72 cm/s, without mitral regurgitation and no history of diabetes were significantly associated with PFO. (Table 2).

**Table 2:** Univariate and multivariate analysis of the predictors of PFO

	Univariate		Multivariate	
	OR (95%CI)	*P* Value	OR (95%CI)	*P* Value
**Echocardiographic Characteristic**				
ARd >33 mm	1.99 (1.13–3.5)	0.016	2.28 (1.18–4.39)	0.013
Em <72 cm/s	2.26 (1.22–4.18)	0.009	2.23 (1.08–4.59)	0.029
Without mistral regurgitation	1.94 (1.09–3.44)	0.023	2.11 (1.14–3.93)	0.017
**Patient Characteristic**				
No history of hypertension			0.88 (0.45–1.71)	0.71
No history of diabetes			2.66 (1.1–6.43)	0.029
No history of stroke or TIA			0.89 (0.39–2.03)	0.784
Nonsmoker			1.43 (0.73–2.79)	0.291
Cortical infarct on imaging			1.22 (0.66–2.27)	0.519
**Age (y)**				
18–29			1.04 (0.74–1.45)	0.816
30–39			1.07 (0.76–1.49)	0.69
40–49			1.24 (0.81–1.91)	0.31
50–59			1.39 (0.74–2.59)	0.298
69–69			0.96 (0.26–3.54)	0.96
* ≥ 70			NA	NA

*PFO* patent foramen ovale, *OR* Odds ratio, *ARd* aortic root diameter, *Em* peak E wave velocity

* All the patients were younger than 70y in our study

A 4-points score system (including without mitral regurgitation, Em < 72 cm/s, ARd >33 mm and no history of diabetes, MEAD) was derived to predict PFO. (Table 3) Based on the similarity of the odds ratios (OR), we assigned a single point for each of the 4 predictors in MEAD score. (Table 2) MEAD score predicted PFO with an AUC of 0.67 (95%CI 0.6–0.75, *p* < 0.001). (Fig. 3, in Supplement) The predictive values of each significance and score were list in Table 4. MEAD score ≥ 3 was the best threshold to predict PFO with an accuracy of 0.64 (95% CI 0.57–0.7), a sensitivity of 0.65 (95% CI 0.53–0.75) and a specificity of 0.63 (95% CI 0.55–0.71). MEADs ≥2 predicted PFO with the highest sensitivity of 0.91 (95%CI 0.82–0.96), while MEADs =4 predicted PFO with the highest specificity of 0.92 (95%CI 0.87–0.96).

**Table 3:** MEAD score

Characteristic	Points
Without **M**itral regurgitation	1
**E**m < 72 cm/s	1
**A**Rd > 33 mm	1
No history of **D**iabetes	1
**Total score** (sum of individual points)	4

*Em* peak E wave velocity, *ARd* aortic root diameter

**Table 4:** AUC, sensitivity, specificity, PPV and NPV of each predictor and each threshold of MEAD score for the detection of PFO

	AUC (95% CI)	Sensitivity (95% CI)	Specificity (95% CI)	PPV (95% CI)	NPV (95% CI)
*Predictive power of each predictor*					
Without mitral regurgitation	0.58 (0.5–0.65)	0.65 (0.53–0.74)	0.5 (0.42–0.59)	0.42 (0.33–0.51)	0.72 (0.62–0.8)
Em <72 cm/s	0.58 (0.51–0.66)	0.75 (0.64–0.84)	0.42 (0.33–0.5)	0.42 (0.33–0.5)	0.75 (0.64–0.84)
ARd >33 mm	0.58 (0.5–0.66)	0.52 (0.4–0.63)	0.64 (0.55–0.72)	0.45 (0.34–0.55)	0.7 (0.62–0.78)
No history of diabetes	0.54 (0.46–062)	0.87 (0.77–0.93)	0.22 (0.15–0.29)	0.38 (0.31–0.46)	0.76 (0.59–0.87)
*Predictive power of different MEADs thresholds*					
MEADs ≥1*	0.5 (0.42–0.58)	NA	NA	0.35 (0.29–0.42)	NA
MEADs ≥2	0.56 (0.49–0.64)	0.91 (0.82–0.96)	0.22 (0.16–0.3)	0.39 (0.32–0.47)	0.82 (0.66–0.92)
MEADs ≥3	0.64 (0.56–0.72)	0.65 (0.53–0.75)	0.63 (0.55–0.71)	0.5 (0.39–0.6)	0.76 (0.67–0.84)
MEADs =4	0.58 (0.5–0.66)	0.24 (0.15–0.35)	0.92 (0.87–0.96)	0.65 (0.45–0.82)	0.68 (0.61–0.75)

*AUC* aera under curve, *PPV* positive predictive value, *NPV* negative predictive value, *PFO* patent foramen ovale, *ARd* aortic root diameter, *Em* peak E wave velocity

*All patients in our study were with MEADs ≥1

In 10-fold cross validation analysis, the mean AUC value of MEAD score was 0.67 in the prediction of PFO. (Table 1, in Supplement).

## Discussion

We were able to develop a simple score to predict the detection of PFO in ESUS patients. The MEAD score measured with non-contrast TTE could be used to identify high-risk patients for PFO and reduce the use of bubble test. This allows the clinician to easily screen patients at highest risk of PFO for further confirming exams. Moreover, our data addressed the echocardiographic changes of heart structures and functions in PFO patients, which could be useful to explore the potential mechanism of ESUS caused by PFO.

Dilatation of the aortic root may increase the risk of RLS by changing the angulation of the heart in such a way that flow streaming from the inferior vena cava into the right atrium is directed more towards the ostium secundum; thrombotic material is therefore more likely to cross into the systemic circulation, possibly causing a cryptogenic stroke. It has been reported that ARd, marked at the level of the sinuses of Valsalva (34 ± 4 vs 31 ± 3 mm, *p* < 0.01), is larger in PFO patients with cryptogenic stroke than in healthy people [7]. In this study, we compared the ARd in a ESUS patient cohort. All subjects had a homologous profile and our results consisted with previous study, demonstrated a larger median ARd of 34 mm in PFO patients than those without PFO.

E-wave velocity reflects the left atrial (LA)-left ventricle (LV) pressure gradient during early diastole and is affected by alterations in the rate of LV relaxation and LA. Elevated LA pressure is associated with the absence of RLS in AF stroke patients and may prevent opening of a PFO [8]. In our study, patients with PFO had lower Em suggest a decreased LA pressure, may associated with a RLS-related stroke. We explored that Em < 72 cm/s could be a mark in predicting PFO which has never been reported before.

Mitral regurgitation is the most common valvular heart disorder in high-income countries, and its prevalence increases with age [9]. In a large-scale cohort of UK adults with 10 years of follow-up, elevated blood pressure was continuously associated with an increased risk of mitral regurgitation [10]. Considering that the association between high blood pressure and mitral regurgitation is similar with that of arteriosclerosis stroke. ESUS patients with no mitral regurgitation may have fewer atherosclerotic risk factors, however, higher likelihood of PFO.

The RoPE score is a useful tool to assess the likelihood that the PFO is responsible for the event stroke. RoPE score was reported to identify PFO with an AUC of 0.68 [4]. However, RoPE score does not include echocardiographic features which could be more directly associated with PFO than other clinical features. In our cohort, there was no difference of RoPE score or proportion of RoPE >6 between PFO and non-PFO groups. In our study, the AUC of MEAD score in predicting PFO was 0.67, which is comparable to the RoPE score.

TEE and bubble test with TTE or TCD are current diagnostic approach of PFO, however, still not widely used in many hospitals due to their inconvenience. Non-contrast TTE is a convenient examination and routinely given to stroke patients for the assessment of cardiac function. The score system we derived based on non-contrast TTE findings can be easily used. MEADs ≥3 predicted PFO with the highest accuracy of 0.64, MEADs ≥2 predicted PFO with a high sensitivity of 0.91, while MEADs =4 predicted PFO with a high specificity of 0.92. By setting different thresholds, MEAD score can serve as a screening tool or a diagnostic tool, which may help doctors to screen patients for the next TEE or bubble test. It could be feasible to increase the positive rate of the following bubble test and reduce the overconsumption of clinical resources in clinical practice.

Several limitations of the present study need to be underlined. First, it was a single-center retrospective cohort study with a small sample size. Our score was built to estimate the probability of finding a PFO in ESUS patients, however, not to assess the likelihood that PFO is responsible for the stroke. Second, PFO was only diagnosed by the bubble test of TCD, not TEE. Although TEE was considered to be the standard technique for identifying a PFO, some patients were intolerant of this method. Finally, all patients in our study had a stroke. Thus, the differences between those with and without stroke in PFO were not examined. The score was not assessed to predict who will develop an ESUS, which needs to be addressed in further studies.

## Conclusions

MEAD score is a risk score system based on non-contrast TTE parameters and patient characteristic that can easily be used to select patients for bubble test to increase the diagnostic yield of PFO after ESUS and might improve the secondary preventive strategy in order to prevent recurrent ischemic strokes.

## Supplementary Information

**Additional file 1: Figure 1**. ROC curve of aortic root diameter (ARd) for predicting PFO. **Figure 2**. ROC curve of peak E wave velocity (Em) for predicting PFO. **Figure 3**. ROC curve of MEAD score for predicting PFO. **Table 1**. 10-flod cross validation of MEAD score for predicting PFO.

## Data Availability

The datasets generated and/or analysed during the current study are not publicly available due to limitations of ethical approval involving the patient data and anonymity but are available from the corresponding author on reasonable request.

## Abbreviations

TTE: Transthoracic echocardiography
TEE: Transesophageal echocardiography
PFO: Patent foramen ovale
ESUS: Embolic stroke of undetermined source
TCD: Transcranial Doppler
ARd: Aortic root diameter
Em: Peak E wave velocity
Am: Peak A wave velocity
RoPE: Risk of Paradoxical Embolism
MCA: Middle cerebral artery
MBs: Microbubbles
RLS: Right-to-left shunt
LAAPd: Left atria anteroposterior diameters
EDLVd: End-diastolic left ventricle diameters
ESLVd: End-systolic left ventricle diameters
LVEF: Left ventricle ejection fraction
ROC: Receiver operating characteristic
IQR: Interquartile range
