# Supplementary material for: A score of non-contrast transthoracic echocardiography to screen patent foramen ovale in patients with embolic stroke of undetermined source
Source: BMC Neurol. 2022 Feb 4;22:43. doi: 10.1186/s12883-022-02565-w (PMC8815249; doi:10.1186/s12883-022-02565-w)
Supplement: Supplementary file 1 — Additional file 1: Figure 1. ROC curve of aortic root diameter (ARd) for predicting PFO. Figure 2. ROC curve of peak E wave velocity (Em) for predicting PFO. Figure 3. ROC curve of MEAD score for predicting PFO. Table 1. 10-flod cross validation of MEAD score for predicting PFO. [file 12883_2022_2565_MOESM1_ESM.docx]

**SUPPLEMENTAL MATERIAL**

**A score of non-contrast transthoracic echocardiography to screen patent foramen ovale in patients with embolic stroke of undetermined source**

**Contents**

*Figure I. ROC curve of aortic root diameter (ARd) for predicting PFO….………………….…….3*

*Figure II. ROC curve of peak E wave velocity (Em) for predicting PFO…………………..……....4*

*Figure III. ROC curve of MEAD score for predicting PFO.………………………………………….5*

*Table I. 10-flod cross validation of MEAD score for predicting PFO…………………………………….6*

**Figure I. ROC curve of aortic root diameter (ARd) for predicting PFO**


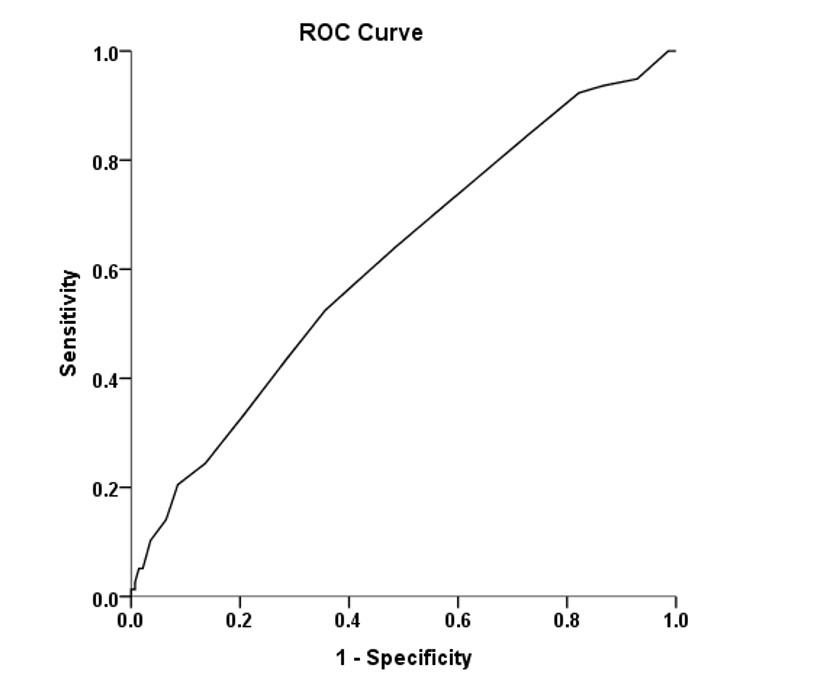


**Figure II. ROC curve of peak E wave velocity (Em) for predicting PFO**


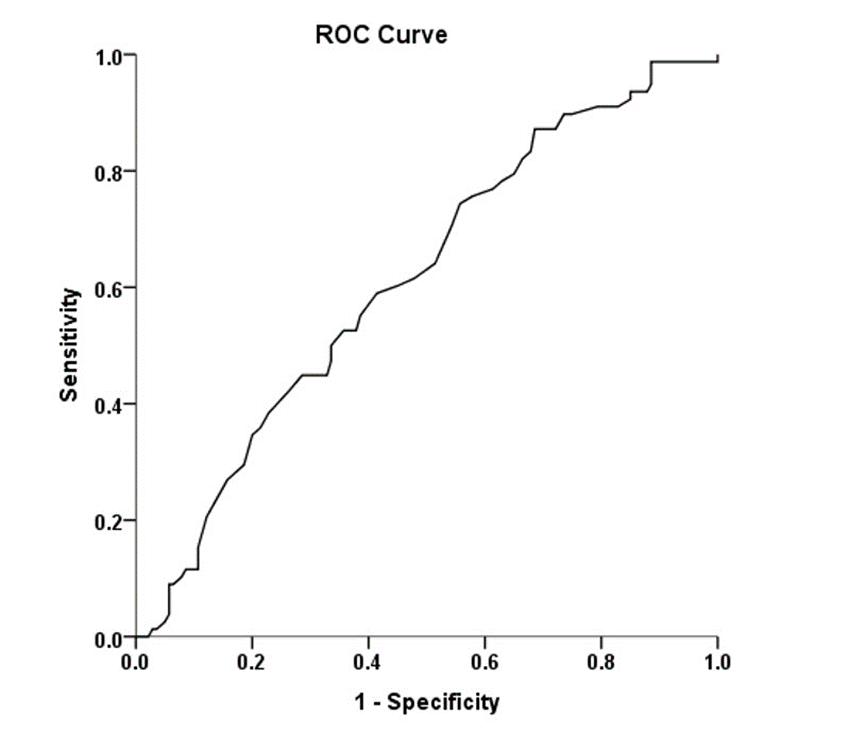


**Figure III. ROC curve of MEAD score for predicting PFO**


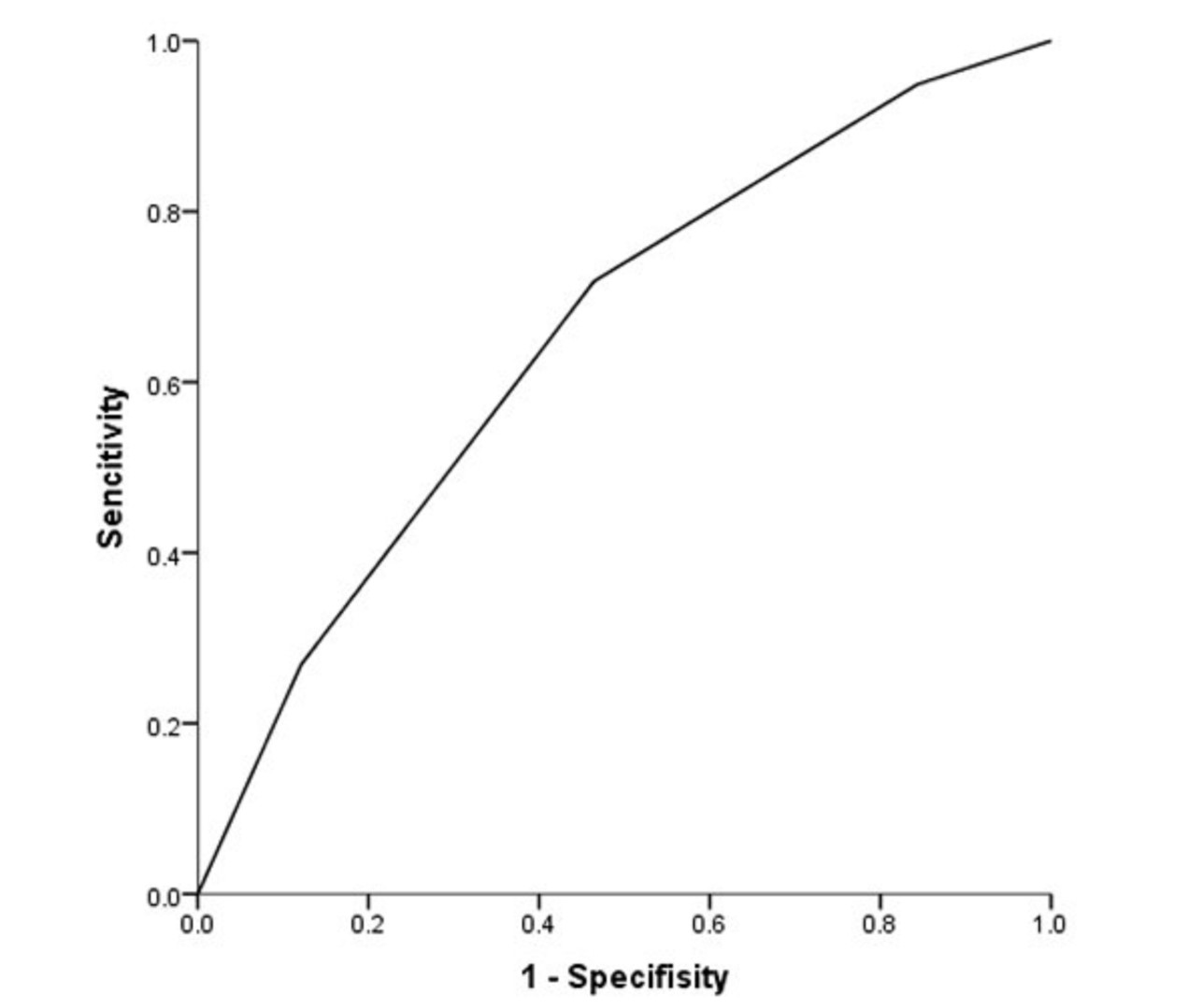


**Table I. 10-flod cross validation of MEAD score for predicting PFO**

| **Sequence** | **AUC value** |
| --- | --- |
| [1] | 0.67 |
| [2] | 0.61 |
| [3] | 0.83 |
| [4] | 0.54 |
| [5] | 0.64 |
| [6] | 0.84 |
| [7] | 0.66 |
| [8] | 0.54 |
| [9] | 0.74 |
| [10] | 0.62 |
| Mean | 0.67 |
